# Quality of life in patients undergoing minimally invasive cardiac surgery: a systematic review

**DOI:** 10.1007/s12055-023-01501-y

**Published:** 2023-04-03

**Authors:** Jade Claessens, Roxanne Rottiers, Jeroen Vandenbrande, Ine Gruyters, Alaaddin Yilmaz, Abdullah Kaya, Björn Stessel

**Affiliations:** 1 Department of Cardiothoracic Surgery, Jessa Hospital, Stadsomvaart 11, Hasselt, Belgium; 2 Faculty of Medicine and Life Sciences, LCRC, UHasselt - Hasselt University, Martelarenlaan 45, 3500 Hasselt, Belgium; 3 Department of Anesthesiology and Perioperative Medicine, Ghent University, Corneel Heymanslaan 10, Ghent, Belgium; 4 Department of Anaesthesiology and Pain Medicine, Jessa Hospital, Stadsomvaart 11, Hasselt, Belgium

**Keywords:** Minimally invasive cardiac surgery, Quality of life, Quality of recovery, SF-36, EQ-5D

## Abstract

**Objective:**

Minimally invasive procedures have been developed to reduce surgical trauma after cardiac surgery. Clinical recovery is the main focus of most research. Still, patient-centred outcomes, such as the quality of life, can provide a more comprehensive understanding of the impact of the surgery on the patient’s life. This systematic review aims to deliver a detailed summary of all available research investigating the quality of recovery, assessed with quality of life instruments, in adults undergoing minimally invasive cardiac surgery.

**Methods:**

All randomised trials, cohort studies, and cross-sectional studies assessing the quality of recovery in patients undergoing minimally invasive cardiac surgery compared to conventional cardiac surgery within the last 20 years were included, and a summary was prepared.

**Results:**

The randomised trial observed an overall improved quality of life after both minimally invasive and conventional surgery. The quality of life improvement in the minimally invasive group showed a faster course and evolved to a higher level than the conventional surgery group. These findings align with the results of prospective cohort studies. In the cross-sectional studies, no significant difference in the quality of life was seen except for one that observed a significantly higher quality of life in the minimally invasive group.

**Conclusions:**

This systematic review indicates that patients may benefit from minimally invasive and conventional cardiac surgery, but patients undergoing minimally invasive cardiac surgery may recover sooner and to a greater extent. However, no firm conclusion could be drawn due to the limited available studies. Therefore, randomised controlled trials are needed.

## Introduction

Although traditional cardiac surgery is performed via median sternotomy, this surgical access route can be associated with major complications. These complications include mediastinitis in 0.3 to 5% of patients, with a mortality rate of 14 to 47% [1]. Since the sternum is divided, sternal instability is another possible complication, which is the leading cause of postoperative morbidity and mortality [2, 3]. These complications can result in a prolonged hospital stay and subsequently a higher healthcare cost [4, 5]. Furthermore, poor cosmetic results of the long midline scar and a significant risk of chronic post-sternotomy pain may negatively affect patient experience [6, 7]. As a result, less invasive surgical access routes to the heart have been investigated [8–17].

Based on physiological parameters and clinical outcomes, including the length of hospital stay and return to work status, it appears that minimally invasive cardiac surgery (MICS) facilitates faster recovery compared to conventional surgery [18–21]. However, these studies often do not assess the quality of recovery (QoR). QoR is a complex phenomenon covering many dimensions of physical, psychological, and social health [22]. It is a subjective experience by the patient without interpretation by a professional [23, 24].

Several instruments have been developed to assess QoR after surgery, such as the QoR-40. This QoR-40 is a good measure for short-term postoperative recovery (1–7 days) [25, 26]. However, quality of life (QoL) questionnaires fulfill the requirements for assessing late QoR (1 month to 1 year) [22, 26]. The 36-item Short-Form health survey (SF-36) and the EuroQol instrument (EQ-5D) are examples of generic instruments. Additionally, disease-specific instruments such as HeartQoL and Seattle Angina Questionnaire can be used. There is no consensus on which questionnaire is best, since disease-specific instruments perform better in certain diseases but limit the comparability with other populations [27]. The most widely used validated generic QoL questionnaire is SF-36 which comprises 36 questions grouped into eight domains: physical functioning, role-physical (limitations on routine activities due to physical problems), pain, general health, role-emotional (limitations on everyday activities due to emotional problems), energy, emotional well-being, and social functioning. Another commonly used generic questionnaire is the validated EQ-5D Questionnaire, developed to analyse QoL on five dimensions (i.e., mobility, self-care, usual activity, pain/discomfort, and anxiety/depression) [22].

This systematic review aims to deliver a detailed summary of all available research investigating the QoR, assessed with QoL instruments in adults undergoing MICS. The aim is to compare the QoR profile after MICS and conventional cardiac surgery.

## Methods

This systematic review is conducted and reported following the Preferred Reporting Items for Systematic Reviews and Meta-Analyses (PRISMA) checklist. A review protocol was published in the PROSPERO register (http://www.crd.york.ac.uk/PROSPERO) in December 2019 with registration number CRD42020163093.

### Search strategy

PubMed, EMBASE, and Cochrane Library databases were searched for relevant articles between December 31, 2019, and December 31, 2020. Articles published before 1999 were excluded. The search was limited to articles written in English.

The appropriate Medical Subject Headings (MeSH) were determined using pilot searches. The following keywords were used: cardiac surgical procedures (MeSH terms), aortic valve/therapy (MeSH terms), mitral valve/therapy (MeSH terms), coronary artery bypass (MeSH terms), Aortic root replacement (MeSH terms), Minimally invasive surgical procedures (MeSH terms), Totally endoscopic (all fields), Health-related Quality of life (all fields), SF-36 (all fields), and EQ-5D (all fields). The final search algorithm was established using Boolean logic operators (“AND” and “OR”).

### Eligibility criteria

We included all published randomised controlled trials (RCTs), cohort studies, and cross-sectional studies assessing the QoR assessed through the QoL in patients undergoing MICS compared to conventional cardiac surgery. We considered studies if participants were adults undergoing specific types of MICS (aortic valve replacement, coronary artery bypass graft, mitral valve repair or replacement, and aortic root replacement) and if QoR has been assessed with SF-36 or EQ-5D. Exclusion criteria included reviews, abstracts, case reports, editorials, retrospective studies, paediatric patients, or no MICS group.

The primary outcome was defined as the QoR measured with SF-36 or EQ-5D at various time points after MICS compared to conventional surgery. Secondary outcomes included clinical endpoints after MICS.

### Data collection and extraction

#### Selection of studies

First, two authors (JC, RR) independently screened article titles based on the exclusion and inclusion criteria. Irrelevant titles were excluded; discrepancies were resolved by discussion with a third party (BS). Next, abstracts of potentially relevant articles were subsequently assessed, and non-relevant articles were excluded. Lastly, the full-text manuscripts of the remaining articles were evaluated. A hand check of reference lists of included studies was performed to identify additional relevant articles.

#### Data extraction and management

Two independent reviewers (JC, RR) performed data extraction and quality assessment of relevant studies using a standard data extraction form. Disagreements were resolved after discussion and consensus by requesting a third reviewer (BS).

#### Assessment of the study quality

The quality assessment tool for controlled intervention studies and observational cohort and cross-sectional studies from the National Institutes of Health was used [28]. This tool includes 14 criteria with a final rating scale of poor, fair, and good. According to their importance, these criteria were sorted to make the articles’ ratings as transparent as possible based on the guidance document. Three levels were used: “primary” when criteria are considered crucial as part of a qualitative study; “secondary” when criteria are not crucial, but if not met, the risk of bias is significantly increased; “tertiary” when not meeting these criteria are not considered to increase the risk of bias. A detailed description of this sorting can be found in the addendum.

In studies where all primary criteria are met, and a maximum of two of the secondaries are not met, a rating of “good” is provided. When a study does not meet the maximum of the primary criteria, and a maximum of two of the secondary criteria are not met, it is assessed as a “fair” study. A “poor” study is identified when more than one of the primary criteria and more than two of the secondary criteria are not met. Although these cross-sectional studies could be considered “good”, we decided to cap the rating of these studies at “fair” as these studies’ design inherently causes less powerful results than controlled interventions or observational cohort studies.

JC and RR assessed the quality of the articles independently. Variability and discrepancies were resolved by discussion with a third party (BS).

### Statistical analysis

Due to enormous heterogeneity in the study population and MICS types included, a meta-analysis could not be performed. Study findings are documented in the form of a “Summary of Findings” table.

## Results

### Study selection

**Fig. 1:**
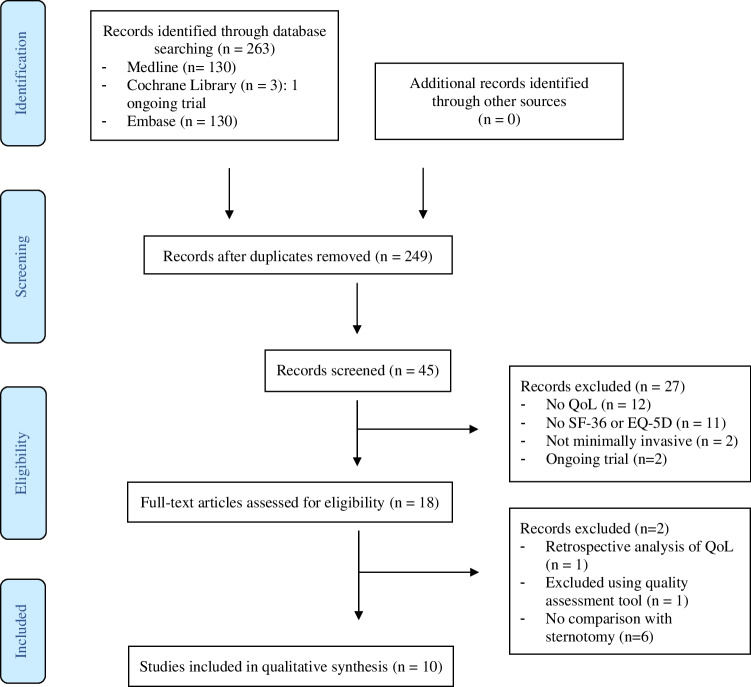
PRISMA flowchart. Abbreviations: QoL, quality of life; SF-36, Short-Form 36; EQ-5D, European Quality of Life 5 dimensions

The search results are presented in a PRISMA flow chart in Fig. 1. After removing the duplicates, 249 records were obtained out of 263 items. A total of 231 manuscripts were excluded based on title and abstract. The number of full-text articles assessed for eligibility was 18. Of these 18 studies, 10 were included in the qualitative synthesis.

### Study characteristics

Two studies focused on coronary artery bypass grafting (CABG), while valve procedures were performed in the other eight studies. Characteristics of all studies are listed in Tables 1 and 2. Only Nasso et al. conducted a randomised trial. Three prospective observational cohort studies assessed QoL before surgery at different follow-up moments [7, 29, 30]. Six studies were performed in a cross-sectional manner [31–36].

**Table 1:** Summary of findings of the included studies regarding coronary artery bypass grafting

Authors	Type of surgery	Sample size (*n*)	Mean age and gender	Country	Study design and time points	Measure of QoL	Study quality	Key findings
Bonaros et al	TECAB (robotic)	MI: *n* = 55 Sternotomy: *n* = 56	MI: 60.2 ± 6 years; 78% males Sternotomy: 64.2 ± 7.3 years; 87.5% males	USA	Prospective cohort T1: admission; T2: 1 month; T2: 3 months; T3: 6 months	SF-36	Good	Physical function significantly improved in the total study population, but was more prominent in the TECAB group. One month after the surgery, TECAB patients had equivalent pain scores as before the surgery. Besides, the improvement of pain symptoms was significantly slower in CABG patients
Ezelsoy et al	MIDCAB (robotic)	MI: *n* = 50 Sternotomy: *n* = 50	MI: 57.4 ± 11.29 years; 52% males Sternotomy: 62.18 ± 6.74 years; 44% males	Turkey	Cross-sectional QoL:4.8 ± 1.9 years after surgery	SF-36	Poor	All domains scored significantly higher in the MIDCAB group compared to the conventional CABG

Abbreviations: *CABG*, coronary artery bypass graft; *MI*, minimally invasive; *MIDCAB*, minimally invasive direct coronary artery bypass; *QoL*, quality of life; *SF-36*, Short-Form 36; *TECAB*, totally endoscopic coronary artery bypass

**Table 2:** Summary of findings of the included studies regarding valve surgery

Authors	Type of surgery	Sample size (*n*)	Mean age and gender	Country	Study design and time points	Measure of QoL	Study quality	Key findings
Nasso et al	Mitral valve repair	MI: *n* = 80 Sternotomy: *n* = 80	MI: 53.9 ± 10.6 years; 57.5% males Sternotomy: 54.3 ± 10.5 years; 56.3% males	Italy	Randomised trial T1: admission; T2: 6 months; T3: 12 months; T3: 2 years; T4: 3 years	SF-36	Good	Physical functioning, role limitations, general health, and energy were significantly better in the MI group 6 months after surgery, indicating faster QoL re-establishment after MI than sternotomy
Moscarelli et al	Valve procedures	MI: *n* = 50 Sternotomy: *n* = 50	MI: 73 ± 10.2 years; 44% males Sternotomy: 69 ± 11.5 years; 76% males	Italy and UK	Prospective cohort T1: admission; T2: 3 months; T2: 6 months; T3: 12 months	EQ-5D	Good	The immediate postoperative QoL was superior in the MI group. However, 6 and 12 months after the surgery, no difference in QoL was observed
Piarulli et al	Valve procedures	MI: *n* = 39 Sternotomy: *n* = 48	MI: 68 ± 8.4 Sternotomy: 64.2 ± 16.7	Italy	Prospective cohort T1: admission; T2: 6 months	EQ-5D	Good	The QoL was significantly better in the MI group compared to the sternotomy patients
Huang et al	Mitral valve surgery	MI: *n* = 78 Sternotomy: *n* = 85	MI: 51.19 ± 11.87; 55.1% males Sternotomy: 51.69 ± 11.69; 64.7% males	China	Cross-sectional QoL: 3 months	SF-36	Fair	Pain and mental health were higher in the MI group compared to sternotomy patients. Other domains were not significantly different
Lange et al	Mitral valve repair	MI: *n* = 501 Sternotomy: *n* = 244	MI: 55 ± 12 years; 72% males Sternotomy: 64 ± 15 years; 61% males	Germany	Cross-sectional QoL: 1.6 ± 1.6 years	SF-36	Fair	The physical component score was significantly higher in the MI group
Detter et al	Aortic valve replacement	MI: *n* = 70 Sternotomy: *n* = 70	MI: 64.3 ± 1.3 years; 57.1% males Sternotomy: 64.2 ± 1.3 years; 57% males	Germany	Cross-sectional QoL: 34.0 ± 10.3 months	SF-36	Fair	No significant differences in QoL between the MI group and sternotomy group
Wachter et al	Aortic root replacement	MI: *n* = 117 Sternotomy: *n* = 75	MI: 56.5 ± 13.6 years; 81.2% males Sternotomy: 64.8 ± 11.6 years; 69.3% males	Germany and UK	Cross-sectional QoL: 2.9 ± 1.3 years	SF-36	Fair	No significant differences in QoL between the MI group and sternotomy group
Franke et al	Aortic valve replacement	MI: *n* = 78 Sternotomy: *n* = 58	MI: 50.4 ± 7.9 years; 82% males Sternotomy: 49.4 ± 10.0 years; 69% males	UK	Cross-sectional QoL: At least 6 months	SF-36	Poor	Physical functioning and role limitations due to physical health were significantly better in the MI group

Abbreviations: *EQ-5D*, European Quality of Life 5 dimensions; *MI*, minimally invasive; *QoL*, quality of life; *SF-36*, Short-Form 36

### Assessment of the study quality

Based on the National Institutes of Health (NIH) quality assessment tool, four studies were rated as “good” [7, 29, 30, 37], while four cross-sectional studies received the rating of “fair” [31, 32, 34, 35] (Table 3). Two studies were identified as “poor” due to methodological issues, including the small sample size, low participation rate, and no description of the patient selection process or follow-up [33, 36].

**Table 3:**
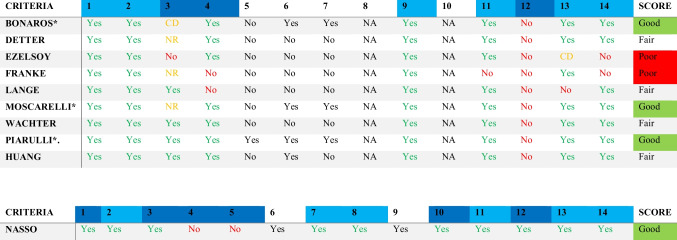
The final rating of the observational cohort and cross-sectional studies

Light blue, primary criteria; dark blue, secondary criteria; others, tertiary criteria

^*^Observational cohort studies; *CD*, cannot determine; *NR*, not reported; *NA*, not applicable

### Study outcomes

The available data regarding the QoL of all included studies are presented in Tables 4 and 5.

**Table 4:** Available data regarding the Short-Form 36 (SF-36) Questionnaire after coronary artery bypass grafting (CABG)

		Bonaros et al			Ezelsoy et al		
		Robotic TECAB	CABG	***p*-**value	Robotic-assisted MIDCAB	CABG	***p*-**value
Baseline: admission	Physical functioning						
	Pain	67.3 ± 31.4					
	General health						
	Role emotional						
	Energy/fatigue						
	Emotional well-being						
	Social functioning						
One month postop	General health	72.0 ± 9.2	60.0 ± 11.9	0.047			
Three months postop	Physical functioning						
	Pain	94.0 ± 8.4	67.3 31.4	0.006			
	General health						
	Role emotional						
	Energy/fatigue						
	Emotional well-being						
	Social functioning						
Available follow**-**up	Physical functioning				80.44 ± 19.85	63.4 ± 19.82	0.008
	Role physical				82.79 ± 55.11	45.42 ± 55.63	< 0.0001
	Pain				74.22 ± 23.57	53.4 ± 27.12	< 0.0001
	General health				63.89 ± 22.44	45.49 ± 21.19	0.001
	Role emotional				81.36 ± 35.67	55.63 ± 47.71	0.003
	Energy/fatigue				55.56 ± 24.76	45.24 ± 22.16	0.02
	Emotional well-being				71.56 ± 21.23	61.24 ± 22.43	0.007
	Social functioning				84.51 ± 21.78	65.4 ± 25.78	0.005

Abbreviations: *CABG*, coronary artery bypass grafting; *MIDCAB*, minimally invasive direct coronary artery bypass; *TECAB*, totally endoscopic coronary artery bypass

**Table 5:** Available data regarding the Short-Form 36 (SF-36) and European Quality of Life 5 Dimensions (EQ-5D) Questionnaire after valve surgery

			Nasso et al			Moscarelli et al			Piarulli et al		
			MICS	Sternotomy	*p*-value	MICS	Sternotomy	*p*-value	MICS	Sternotomy	*p*-value
Baseline	SF-36	Physical functioning	53.8 ± 5	54.4 ± 6							
		Role limitation	52.6 ± 8.1	52.1 ± 7.6							
		Pain									
		General health	51.3 ± 6.2	51.9 ± 7.6							
		Energy/fatigue	60.3 ± 3.9	59.6 ± 4.3							
		Emotional well-being	79.4 ± 9.3	76.2 ± 6.1							
		Social functioning	75.7 ± 5.5	76.2 ± 5.4							
	EQ-5D	VAS				64.1 ± 15.7	69.4 ± 13.1	< 0.001			
		Index value				0.6 ± 0.2	0.5 ± 0.1	0.2			
Three months postop	EQ-5D	VAS				64.6 ± 12.8	62.3 ± 11.2	0.002			
		Index value				0.6 ± 0.2	0.4 ± 0.2	< 0.001			
Six months postop	SF-36	Physical functioning	64.9 ± 8.3	53.1 ± 7	< 0.001						
		Role limitation	67.4 ± 9.1	51.7 ± 6.6	< 0.001						
		Pain									
		General health	70.2 ± 8.7	52.3 ± 7	< 0.001						
		Energy/fatigue	80.4 ± 9.3	76.4 ± 8.5	< 0.001						
		Emotional well-being	79.4 ± 9.3	79.1 ± 8.8	0.56						
		Social functioning	73.4 ± 6.8	71.9 ± 7	0.13						
	EQ-5D	VAS							80.8 ± 14.9	70.8 ± 19.6	0.03
		Index value							0.78 ± 0.25	0.52 ± 0.49	0.01
Avalable follow-up	SF-36	Physical functioning	79.1 ± 9.2	79.7 ± 8.5							
		Role limitation									
		Pain									
		General health	82.9 ± 9.7	84.2 ± 8.7							
		Energy/fatigue	79.8 ± 8.6	78.8 ± 8.2							
		Emotional well-being	82.4 ± 9.3	81.5 ± 8.9							
		Social functioning	84.2 ± 7	81.5 ± 8.9							
			Huang et al			Lange et al			Detter et al		
			MICS	Sternotomy	*p*-value	MICS	Sternotomy	*p*-value	MICS	Sternotomy	Three-value
Three months postop	SF-36	Physical functioning	77.75 ± 8.07	77.46 ± 8.08	0.82						
		Role physical	71.15 ± 15.12	68.82 ± 15.39	0.33						
		Role emotional	64.14 ± 17.86	62.18 ± 13.97	0.24						
		Pain	77.05 ± 14.78	70.12 ± 12.85	0.24						
		General health	65.13 ± 13.31	63.29 ± 1.51	0.347						
		Energy/fatigue	64.17 ± 11.99	63.29 ± 11.51	0.64						
		Emotional well-being	74.62 ± 13.63	68.42 ± 17.95	0.015						
		Social functioning	71.71 ± 12.20	70.38 ± 11.87	0.48						
Available follow-up	SF-36	Physical functioning							68.5 ± 29.8	69.7 ± 29.1	ns
		Role physical							56.9 ± 45.1	61.5 ± 43.6	ns
		Role emotional							80.9 ± 36.6	75.8 ± 44.3	ns
		Pain							87.5 ± 22.4	78.9 ± 31.4	ns
		General health							63.4 ± 18.0	62.4 ± 21.7	ns
		Energy/fatigue							60.7 ± 24.3	63.7 ± 25.4	ns
		Emotional well-being							74.2 ± 20.5	75.2 ± 25.0	ns
		Social functioning							85.4 ± 21.5	80.3 ± 31.8	ns
		Physical component score				47.7 ± 7.7	46.3 ± 10.6	0.45			
		Mental component score				50.2 ± 9.2	49.5 ± 11.0	0.69			
			Wachter et al			Franke et al					
			MICS	Sternotomy	*p*-value	MICS	Sternotomy	*p*-value			
Available follow-up	SF-36	Physical functioning	82.8	76.8	0.051	85.1 ± 18.4	76.8 ± 21.6	0.027			
		Role physical	74.2	69.9	0.778	79.2 ± 35.8	63.0 ± 41.3	0.024			
		Role emotional	80.1	72.2	0.180			0.843			
		Pain	85.2	80.2	0.216			0.120			
		General health	67.5	65.6	0.448			0.192			
		Energy/fatigue	60.0	57.3	0.311			0.073			
		Emotional well-being	72.9	72.0	0.742			0.323			
		Social functioning	85.4	81.6	0.129			0.098			

Abbreviations: *EQ-5D*, European Quality of Life 5 dimensions; *MICS*, minimally invasive cardiac surgery; *SF-36*, Short-Form 36 Questionnaire; *VAS*, visual analogue scale

### Domains of SF-36

#### Physical functioning

##### CABG

Bonaros et al. reported a more prominent physical functioning improvement after MICS (robotic totally endoscopic coronary artery bypass (TECAB)) than sternotomy. Three months after MICS, it reached almost 100% [30]. Regarding robotic-assisted minimally invasive direct coronary artery bypass (MIDCAB), the cross-sectional study reported significantly better physical functioning after MICS (MICS: 80.44 ± 19.85, sternotomy: 63.4 ± 19.82, *p* = 0.008; Table 4) [33].

##### Valve surgery

In the sole included RCT looking at mitral valve repair, physical functioning improved significantly in the MICS group compared to conventional cardiac surgery patients after 6 months (MICS: 64.9 ± 8.3, sternotomy: 53.1 ± 7, *p* < 0.001; Table 5) [37]. Additionally, a cross-sectional study reported significantly better physical functioning after MICS for the Ross procedure (MICS: 85.1 ± 18.4, sternotomy: 76.8 ± 21.6, *p* = 0.027; Table 5 [36]). In contrast, three other studies regarding valve surgery did not observe a significant difference between MICS and sternotomy [32, 34, 35].

#### Role limitations

##### CABG

The role-emotional scores significantly improved after both robotic TECAB and conventional CABG [30]. On the other hand, a significantly better role-physical was seen after robotic-assisted MIDCAB compared to conventional CABG in the cross-sectional study (MICS: 82.79 ± 55.11 sternotomy: 45.42 ± 55.63, *p* < 0.001; Table 4) [33].

##### Valve surgery

The mitral valve repair RCT observed a significantly better role-emotional score in the MICS group compared to sternotomy after 6 months (MICS: 67.4 ± 9.1, sternotomy: 51.7 ± 6.6, *p* < 0.001; Table 5) [37].

The role-physical score was significantly better in the MICS group compared to conventional cardiac surgery in the Ross procedure (MICS: 79.2 ± 35.8, sternotomy: 63.0 ± 41.3, 69, *p* = 0.024; Table 5 [36]). The other studies reporting on this topic observed no significant difference [32, 34, 35].

#### Pain

##### CABG

After robotic TECAB, patients stated equivalent pain scores 1 month after surgery compared to baseline. They reported significantly less pain (a higher score) at 3 months after MICS compared to baseline (baseline: 67.3 ± 31.4, 3 months: 94 ± 8.4, *p* = 0.006; Table 4). Patients who received a sternotomy proclaimed more pain (a lower score) in comparison to MICS patients after 3 months (MICS: 94 ± 8.4, sternotomy: 79.0 ± 21.0, *p* = 0.037; Table 4) [30]. Significantly less pain (a higher score) was noted in the robotic-assisted MIDCAB patients (MICS: 74.22 ± 23.57, sternotomy: 53.4 ± 27.12, *p* < 0.001; Table 4) [33].

##### Valve surgery

A cross-sectional study noted significantly less pain (a higher score) in the MICS group after mitral valve surgery (MICS: 77.05 ± 14.78 and sternotomy: 70.12 ± 12.58; Table 5) [34]. However, all other studies regarding valve surgery observed no significant improvement [32, 35, 36]. The RCT did not report anything regarding pain [37].

#### General health

##### CABG

Bonaros et al. reported a significantly better general health score than sternotomy 1 month after robotic TECAB (MICS: 72 ± 9.2, sternotomy: 60 ± 11.9, *p* = 0.047; Table 4) [30]. The cross-sectional study reported superior long-term general health after MICS in robotic-assisted MIDCAB (MICS: 63.89 ± 22.44, sternotomy: 45.49 ± 21.19, *p* = 0.001; Table 4) [33].

##### Valve surgery

The RCT, looking at mitral valve repair, found significantly better general health in the MICS group 6 months after surgery (MICS: 70.2 ± 8.7, sternotomy:52.3 ± 7, *p* < 0.001; Table 5) [37]. In contrast, in several cross-sectional studies, no significant difference in general health was observed between MICS and the control group after valve surgery [32, 34–36].

#### Energy

##### CABG

The energy score improved after MICS as well as after conventional cardiac surgery after 3 months post robotic TECAB [30], but was significantly better in the MICS group in the cross-sectional study investigating robotic-assisted MIDCAB (MICS: 55.56 ± 24.76, sternotomy: 45.24 ± 22.16, *p* = 0.02; Table 4) [33].

##### Valve surgery

The energy levels were significantly in favour of the MICS group after 6 months post mitral valve repair (MICS: 80.4 ± 9.3, sternotomy: 76.4 ± 8.5, *p* < 0.001; Table 5) [37]. In contrast, four other cross-sectional studies observed no significant difference [32, 34–36].

#### Emotional well-being

##### CABG

No significant difference was seen between MICS and sternotomy after CABG at 6 months postoperatively (MICS: 79.4 ± 9.3 and sternotomy: 79.1 ± 8.8, respectively, *p* = 0.56; Table 4) [30]. However, an improvement in emotional well-being was observed after robotic-assisted MIDCAB compared to sternotomy (MICS: 71.56 ± 21.23, sternotomy: 61.24 ± 22.43, *p* = 0.007; Table 4)[33].

##### Valve surgery

The RCT, investigating mitral valve repair, reported no significant difference in emotional well-being between baseline and follow-up after MICS (76.8 ± 7 and 79.4 ± 9.3, respectively; Table 5) [37]. A cross-sectional study observed an improvement in emotional well-being after MICS compared to sternotomy after mitral valve surgery (MICS: 74.62 ± 13.63, sternotomy: 68.42 ± 17.95, *p* = 0.015; Table 5) [34] while other valve-related studies observed no significant difference [32, 35, 36].

#### Social functioning

##### CABG

Social functioning did not differ between MICS and sternotomy after robotic TECAB at 6 months postoperatively (MICS: 73.4 ± 6.8 and sternotomy: 71.9 ± 7, *p* = 0.1; Table 4), nor after robotic-assisted MIDCAB [30, 33].

##### Valve surgery

A significant difference in social functioning could not be detected by the RCT between baseline and follow-up after MICS in mitral valve repair [37]. Additionally, in the other studies, no significant improvement was seen [32, 34–36].

#### Domains of EQ-5D

Two prospective cohort studies reported an overall improved QoL through the EQ-5D Questionnaire after valve procedures. In Moscarelli et al., the immediate postoperative QoL was superior in the MICS group. However, no difference in QoL was observed 6 and 12 months after the surgery between MICS and sternotomy [29]. Secondly, Piarulli et al. observed a significantly better QoL in the MICS group than in the sternotomy patients [7]. The specific QoL data is displayed in Table 5.

#### Clinical outcome

The summary of relevant clinical outcomes of all included studies is presented in Tables 6 and 7.

**Table 6:** Summary of clinical outcomes regarding valve surgery

	CPB time (min)			Cross**-**clamping time (min)			ICU LOS			Ventilation time		
	MI	Control	***p*-**value	MI	Control	***p*-**value	MI	Control	***p*-**value	MI	Control	***p*-**value
Bonaros et al	125.4 ± 42.5	133.7 ± 24.5	ns	76.6 ± 23.9	82.1 ± 21.1	ns	30.6 ± 19.5 h	34.7 ± 16.8 hours	< 0.05	12.3 ± 12.2 h	13.6 ± 16.8 h	ns
Ezelsoy et al							1.3 days	2.1 days	< 0.05			
	In**-**hospital mortality (%)			Hospital LOS (days)			Cardiac**-**related mortality (%)			Reoperation (%)		
	MI	Control	***p*-**value	MI	Control	***p*-**value	MI	Control	***p*-**value	MI	Control	***p*-**value
Bonaros et al	0	0	ns	9.8 ± 6.2	19.2 ± 11.9	0.001						
Ezelsoy et al	0	0	ns	5.62	7.96	< 0.001	0	0	ns			

Abbreviations: *CPB*, cardiopulmonary bypass; *ICU*, intensive care unit; *LOS*, length of stay; *MI*, minimally invasive techniques; *ns*, not significant; *NR*, not reported. Data are presented as mean SD or median (25^th^, 75^th^ percentile)

**Table 7:** Summary of clinical outcomes regarding coronary artery bypass grafting

	CPB time (min)			Cross**-**clamping time (min)			ICU LOS			Ventilation time		
	MI	Control	***p*-**value	MI	Control	***p*-**value	MI	Control	***p***-value	MI	Control	***p***-value
Nasso et al	137 ± 28	102 ± 23	< 0.001	91 ± 22	69 ± 19	< 0.001	2.1 ± 1 days	3.2 ± 1.1 days	0.013	1.2 ± 0.3 days	2.2 ± 0.6 days	0.01
Moscarelli et al	110.7 ± 36	92 ± 25	0.006	73.6 ± 26.9	59 ± 23.7	0.007						
Piarulli et al	122.7 ± 26.1	107.2 ± 25.5	0.001	86.9 ± 21.1	88.7 ± 21	ns						
Huang et al							2.1 ± 1.12 days	2.17 ± 0.82 days	0.16			
Lange et al	121 ± 33	97 ± 29	< 0.001	87 ± 25	70 ± 24	< 0.001	3.6 ± 3.2 days	5 ± 4.8 days	< 0.001	24 (4–256)	34 (4–256)	< 0.001
Detter et al	105 ± 22	84 ± 24	< 0.001	71 ± 15	58 ± 18	< 0.001	1.8 ± 1.2 days	2.3 ± 1.7 days	ns	7.6 ± 5.1	8.8 ± 5.5	ns
Wachter et al	164.9 ± 32.9	167.6 ± 42.5	0.659	131.3 ± 21.9	129.1 ± 25	0.576	1.9 ± 3.6 days	3.2 ± 5.6 days	< 0.001	10.2 ± 21.8 h	26.9 ± 109 h	< 0.001
Franke et al	185 ± 30	177 ± 30	0.118	140 ± 17	151 ± 19	0.001						
	In**-**hospital mortality (%)			Hospital LOS (days)			Cardiac**-**related mortality (%)			Reoperation (%)		
	MI	Control	***p***-value	MI	Control	***p***-value	MI	Control	***p***-value	MI	Control	***p***-value
Nasso et al	2.5	2.5	ns	8.5 ± 4.5	11.6 ± 5	0.02	0	0	ns	2.25	1.25	0.9
Moscarelli et al	0	2	1	8 ± 2	8 ± 4.2	0.8				2	2	1
Piarulli et al	0	0	ns	7.3 ± 3.1	7.8 ± 3.9	NR	0	0	ns	0	0	ns
Huang et al	0	0	ns	5.17 ± 1.75	5.93 ± 1.14	0.77	0	0	ns	0	0	ns
Lange et al				7.9 ± 5.4	9.4 ± 5.1	< 0.001				4.4	7.8	0.24
Detter et al	0	2.9	ns	7.3 ± 4.6	8.5 ± 4.1	ns	2.9	2.9	ns	8.5	8.6	ns
Wachter et al	0.9	2.7	0.562	10.4 ± 5.5	12.9 ± 6.9	< 0.001	0	4	NR	9.4	8	0.510
Franke et al	0	2	NR	11.2 ± 6.3	11.9 ± 7.2	0.523	0	0	ns	17	9	0.172

Abbreviations: *CPB*, cardiopulmonary bypass; *ICU*, intensive care unit; *LOS*, length of stay; *MI*, minimally invasive techniques; *ns*, not significant; *NR*, not reported. Data are presented as mean SD or median (25^th^, 75^th^ percentile

## Discussion

MICS has been developed to reduce surgical trauma caused by sternotomy during conventional cardiac surgery. A good clinical outcome after MICS has already been demonstrated by a lower morbidity and mortality rate [19, 21]. However, patient-reported outcomes have become essential endpoints in medical care. To our knowledge, this is the first systematic review to date to summarise all the available primary research investigating the QoR, assessed with QoL instruments, from the patient’s perspective in adults undergoing MICS.

Both sternotomy and MICS patients benefited from the surgery regarding their QoL. In previous studies assessing the QoL after conventional cardiac surgery, the different scales of the SF-36 Questionnaire significantly improved after surgery. One month after the surgery, the QoL improved after conventional surgery but inadequately. After 1 year, satisfactory QoL results were obtained; after 10 years, even the elderly patients had an improved QoL [38, 39]. According to the studies in this systematic review, MICS patients may recover faster and probably to a greater extent. The only RCT available concluded that physical functioning, role limitations, general health, and energy were significantly better in the MICS group 6 months after mitral valve repair surgery, indicating faster re-establishing of the QoL after MICS than sternotomy. However, most cross-sectional studies observed no significant difference between MICS and conventional surgery, varying from 1 to 5 years after the surgery [31, 33, 35, 36].

Improvement in physical functioning was more prominent in MICS patients, and pain scores of patients undergoing sternotomy improved significantly slower. Overall, general health, as well as energy score, improved in both groups after surgery. However, the MICS patients had an earlier improvement in their general health and indicated that they have significantly more energy than conventional surgery patients. In Gjeilo et al. series, no significant improvement in general health or energy score was seen in conventional cardiac patients after 10 years [39]. Scores on social functioning varied among the various studies. As a result, no firm conclusion could be drawn regarding this subscale. In the study by Pačarić et al., the social functioning did not improve significantly, but was not as low as other subscales at baseline [38]. This same trend is seen in the RCT included in this review [37].

### Limitations

There are several limitations to this systematic review. First, only studies published in English were included, causing a severe risk of language bias. Second, only one RCT comparing QoR after MICS and conventional cardiac surgery could be included making firm conclusions impossible. The lack of RCTs indicates the need for more research regarding the QoL. Without RCTs, a significant selection bias is present. Patients would have been selected based on their age, fragility, number of procedures, preoperative status, and comorbidities to obtain better results. Furthermore, most included studies had a cross-sectional design which does not allow temporal or causal interpretation [40]. No common timepoints were available to calculate summary QoL scores. Finally, the inclusion of studies with different methodologies and different surgeries are significant limitations of this systematic review.

For these reasons, the studies were not directly comparable, and a meta-analysis could not be conducted. However, this comprehensive approach provides a complete overview of the research field and might be helpful for further studies. Future research in QoL after MICS should focus on prospective studies with sufficient sample sizes. A RCT with a MICS arm and a conventional arm would be the best design.

## Conclusion

This systematic review indicates that patients benefit from both MICS and conventional cardiac surgery, but patients undergoing MICS may recover sooner and to a greater extent. However, no firm conclusion could be drawn due to the limited available studies with mainly a cross-sectional design. Therefore, this systematic review’s results should be critically interpreted. More high-quality RCTs comparing QoR after MICS and conventional cardiac surgery should be performed to draw more firm conclusions on differences in QoR.
